# Integrating Diverse Disciplines to Enhance Interprofessional Competency in Healthcare Delivery

**DOI:** 10.3390/healthcare7020075

**Published:** 2019-06-10

**Authors:** Tiffany Champagne-Langabeer, Lee Revere, Mariya Tankimovich, Erica Yu, Robert Spears, Jennifer Lee Swails

**Affiliations:** 1 School of Biomedical Informatics, University of Texas Health Science Center (UT Health), Houston, TX 77030, USA; 2 School of Public Health, University of Texas Health Science Center, Houston, TX 77030, USA; frances.lee.revere@uth.tmc.edu; 3 Cizik School of Nursing, University of Texas Health Science Center, Center, Houston, TX 77030, USA; Mariya.Tankimovich@uth.tmc.edu (M.T.); Erica.Yu@uth.tmc.edu (E.Y.); 4 School of Dentistry, University of Texas Health Science Center, Houston, TX 77030, USA; Robert.D.Spears@uth.tmc.edu; 5 McGovern Medical School, University of Texas Health Science Center, Houston, TX 77030, USA; Jennifer.L.Swails@uth.tmc.edu

**Keywords:** interprofessional education, simulation-based education, standardized patients, medical education, electronic health record

## Abstract

Interprofessional education (IPE) typically involves clinical simulation exercises with students from medical and nursing schools. Yet, healthcare requires patient-centered teams that include diverse disciplines. Students from public health and informatics are rarely incorporated into IPE, signaling a gap in current educational practices. In this study, we integrated students from administrative and non-clinical disciplines into traditional clinical simulations and measured the effect on communication and teamwork. From July 2017–July 2018, 408 students from five schools (medicine, nursing, dentistry, public health, and informatics) participated in one of eight three-hour IPE clinical simulations with Standardized Patients and electronic health record technologies. Data were gathered using a pre-test–post-test interventional Interprofessional Collaborative Competency Attainment Survey (ICCAS) and through qualitative evaluations from Standardized Patients. Of the total 408 students, 386 (94.6%) had matched pre- and post-test results from the surveys. There was a 15.9% improvement in collaboration overall between the pre- and post-tests. ICCAS competencies showed improvements in teamwork, communication, collaboration, and conflict management, with an average change from 5.26 to 6.10 (*t* = 35.16; *p* < 0.001). We found by creating new clinical simulations with additional roles for non-clinical professionals, student learners were able to observe and learn interprofessional teamwork from each other and from faculty role models.

## 1. Introduction

Academic research examining interprofessional education (IPE) has primarily centered around the clinical aspects of healthcare, and including students from nursing and medicine [1,2]. However, since healthcare is transitioning to patient-centered team-based care [3], there is a critical gap that students from allied and public health disciplines (such as healthcare and management or informatics) are rarely incorporated into IPE simulation exercises. Students in public and allied health fields are uniquely trained to provide information on different aspects of care (such as community programs, payment systems, and population health). Dental students are trained in screening procedures for the prevention of disease and treatments which are complementary to overall general health. Similarly, by integrating students who specialize in informatics and health technologies, clinical students have the opportunity to learn about electronic health records (EHR); and equally, informatics students are afforded the opportunity to see the full spectrum of patient-centered care and usability of health information technology. Working together, IPE affords students from diverse healthcare backgrounds the opportunity to learn from each other in an interactive peer environment, which is superior when compared to an online only or lecture-based environment [4]. A structured, interprofessional setting which allows diverse students to learn together has also been shown to improve the climate and capacity for teamwork [5,6].

A core benefit of IPE is to enhance communication among providers who work together with a common goal to improve patient care. The Association of American Medical Colleges (AAMC) requires participation as a member of an interprofessional team as an Entrustable Professional Activity (EPA) [7]; while the American Association of Colleges of Nursing (AACN) places a strategic focus on IPE to improve innovation within the discipline and within the field of healthcare [8]. Innovations are stimulated by diverse perspectives, academic backgrounds, and require strong skills in teamwork, leadership, and communication [9,10]. When students collaborate with others outside their area of expertise, creative thinking skills are enhanced as well as the students’ ability to generate solutions through teamwork [11]. Competencies gained through IPE exercises and case studies have been an effective means to improve diplomacy, policy, and quality outcomes in both undergraduate nursing students [12] and graduate nursing, public health, and physician assistant students [13].

Enhancing collaboration remains a vital objective, yet communication among providers has become multi-faceted and complex from a technological perspective [14]. We posit that diverse teams of clinical and non-clinical administrative students who participate in interprofessional education simulations will improve their level of communication and collaboration. The objective of this study was to evaluate the change in pre- versus post- collaboration scores for students who participated in an IPE simulation involving nursing, medicine, dentistry, public health, and informatics.

## 2. Materials and Methods 

### 2.1. Simulation 

Prior research has shown simulation as an effective pedagogical tool to deliver both IPE and EHR training [15,16]; further, clinicians who trained using EHR through simulation techniques significantly enhanced their safety and quality scores related to EHR usage [17]. Providing students an opportunity to utilize these skills in a structured IPE training environment may be critical to their later success when caring for patients [18,19,20].

This study describes a comprehensive approach and related outcomes to developing an IPE simulation aimed at integrating students from clinical backgrounds (medicine, nursing, dentistry) with students from administrative and non-clinical areas (public health and informatics). The two case-based IPE simulations included students from five schools within a large academic medical center and assessed the effect of the training on students’ communication and teamwork skills. As there are limitations to assessing competencies in simulation-based training, cases were designed using prior research on established conceptual frameworks [21].

### 2.2. Research Design

This study utilized a pre-test–post-test quasi-experimental design [22] and was held in a clinical simulation center with Standardized Patients (SP) and adjacent classrooms equipped with EHRs. The study was conducted in a large, academic medical center comprised of five schools for health professionals (nursing, medicine, dentistry, public health, biomedical informatics). Both convenience and purpose sampling were used to recruit students from each school.

### 2.3. Participants

Clinical students participated as part of their required coursework and received a passing grade for participation. They included those from the medical, dental, and nursing schools (both undergraduate and graduate level nurse practitioners were included). Students from administrative and non-clinical areas were from the schools of public health and biomedical informatics. Non-clinical students participated in the simulation as an optional, experiential exercise. Medical students were in their third and fourth years of undergraduate medical education; students from the nursing school included both Bachelor of Science in Nursing (BSN) third- and fourth-year students and those in any year of the Doctor of Nursing Practice (DNP) degree program. Students from the dental school were from any year in the Doctor of Dental Surgery (DDS) program. Public health students were enrolled in either a Master of Public Health (MPH) or Doctor of Philosophy (PhD); and informatics students were enrolled in either a Master of Science (MS) or Doctor of Philosophy (PhD). Students from the school of public health were from specializations in management, policy, and community health and were from any year in their program. Students from the school of biomedical informatics were enrolled in classes on campus (not online only) and were from any year in their program. Although many of the non-clinical and administrative students had worked in hospitals, none had prior IPE training prior to this simulation. Teams consisted of five members each from varied clinical and professional backgrounds when possible, playing the roles of a doctor, nurse, dentist, quality officer, and chief information officer. When the matching student for the role was unavailable (such as a non-clinical or dental student), a medical or nursing student participated as the substitute and assumed the missing role.

### 2.4. Case Development

A team of faculty from each of the five schools met from February 2017–April 2017 and created two realistic cases centered on aspects of patient safety, prescription drug dosing, communicating a medical error, and discovering a problem hidden in the EHR. The Ambulatory Case described an elderly woman with osteoporosis who recently lost her employer-based insurance. She presented to a student-run free clinic with dental pain and requested a medication refill; however, during her examination, an X-ray was delivered which showed a compression fracture with neurologic compromise. Students were to recognize her condition required immediate hospitalization and were expected to account for her lack of financial resources when deciding on the best plan of action. The Inpatient Case required the students to work together to discover a medical error in the electronic record. The patient in this case was admitted for endocarditis following a dental cleaning. His weight was erroneously recorded in the EHR in kilograms instead of pounds; and he received an overdose of antibiotic, which led to an acute kidney injury and the need for further hospitalization. Students were to perform a root cause analysis and create a plan to disclose the error to the patient. They had an opportunity to practice mutual support techniques while interacting with an angry patient and family member. Faculty mentors from each school and simulation center staff went through the cases as a pilot in order to provide the SPs with practice interacting with team members. This exercise also gave the faculty an opportunity to provide feedback, further refine the cases, and rehearse the timing of events. Final simulations were comparable in their degree of difficulty practiced by faculty and simulation staff. Post-simulation on the day of the IPE simulation, faculty members provided an extensive debrief with students and discussed optimal ways of handling each scenario.

### 2.5. Instruments

Four instruments were used in the study: (1) a demographic survey; (2) a pre-test–post-test interventional Interprofessional Collaborative Competency Attainment Survey (ICCAS) [23]; (3) an SP evaluation to measure interprofessional teamwork, modified from the Team Emergency Assessment Measure (TEAM) [24], and (4) open-ended qualitative questions to measure students’ expectations of team-oriented tasks. The demographic survey requested information regarding the students’ school affiliation, gender, age, and prior IPE training. The ICCAS was used in order to evaluate the effect of the simulation on the students’ perception of their own achievement. Items were scored for the ICCAS portion of the survey using a seven-point Likert-scale ranging from 1 = strongly disagree to 7 = strongly agree. The ICCAS measures competencies across five principle domains including: (1) communication, (2) collaboration, (3) role on the team, (4) team-approach, (5) and team functioning. The pre-test–post-test format of the ICCAS was designed by subject matter experts in the field of IPE; and the aim of this survey is to discover the subtle differences in competency attainment before and after an intervention [23]. The IPE faculty created a rubric for the SPs to evaluate the students as team, which was based upon the TEAM survey by Cooper and colleagues [24]. The original TEAM survey was designed for use in assessing trauma and resuscitation teams; however, the authors found the themes of leadership, teamwork, and task management universally acceptable and modified the tool for a non-emergent environment [24]. The final SP rubric included questions regarding team communication, adaptability to changing situations, ability to respond professionally, respect for the patient, and respect for peers on the team. Open-ended questions were asked regarding the students’ prior IPE training, learning aspirations, and also cued free text responses to questions including: “Do you like to take action or wait for the opinions of others?” and “Do you think it is important to stick to your own decisions?” and “Do you prefer working alone or part of a group?”. After the cases, the students were asked “What did you learn from this activity that you can apply to your discipline?”, “Do you ask others for help?” and “How do you negotiate with others who have different views?”. Students had a free-text entry where they could also describe any recommendations for improvement in the exercise.

### 2.6. Data Collection Procedures

Data were collected from eight three-hour IPE simulations which occurred during the months of July 2017 until July 2018. One week prior to the simulation, students were given access to an online course which had content specific to the skills for the TeamSTEPPS program [25]. The Team Strategies and Tools to Enhance Performance and Patient Safety, or STEPPS as it is commonly known, is a training curriculum that was developedby the Agency for Healthcare Research and Quality [25]. The goals of the program were presented in a video, along with evidence-based research of pre- and post- TeamSTEPPS training [26,27]. Students were required to complete the demographic survey, the pre-test ICCAS survey, and the open-ended qualitative survey one week before the day of the simulation. Students completed all surveys using the institution′s online learning management system.

Outpatient and inpatient simulations were held on different days to accommodate lab space and SP availability. On the day of the simulation, students were divided into teams of five members each from varied clinical and professional backgrounds when possible. Teams were given access to the patient’s EHR which was complete with a past medical history and current information, pre-programmed by the IPE faculty. The students reviewed the patient’s electronic health record and formulated an intentional care plan after assessing the status of the patient. The teams then entered the patient simulation rooms and discussed the plan with their patients (the SPs). The SPs were trained on utilization of the rubric and had the opportunity to practice with faculty before interacting with student teams. At the end of each case, the SPs provided the students with detailed verbal feedback regarding their teamwork skills and individual performance based upon the modified TEAM rubric. Faculty observed each simulation and feedback session synchronously from a viewing room located in the simulation center. The SPs assessed the team’s concerted communication, how they reacted to an unexpected X-ray result, how they shifted their clinical judgement, and how they respected their team members. For the inpatient medical error simulation, the SPs provided feedback regarding team communication when disclosing the medical error to the patient, such as how well the students listened to their colleagues without interruption and how well they supported other members of the team. After each simulation, students had an opportunity to debrief as a group and exercise self-reflection with faculty from interprofessional disciplines to hear best-practice methods and ask questions about their performance. Before being dismissed from the simulation on the day of the event, students were required to complete the post-test ICCAS survey and the open-ended qualitative survey. All results were collected electronically in the institution′s online learning management system.

### 2.7. Ethical Considerations

The University of Texas Health Science Center institutional review board reviewed and exempted this study as educational research. The students provided informed consent to participate in the simulation and no monetary compensation was provided.

### 2.8. Statistical Analysis

Descriptive statistics (frequency analyses) were used to analyze demographic data from student surveys. Data from ICCAS pre-test–post-test scores were extracted, and the paired *t*-test was used to determine statistical significance. Significance was established a priori at *p* < 0.05. Analyses were conducted using IBM SPSS Statistics software, version 24 (Armonk, NY, USA). Qualitative questions were analyzed using thematic synthesis [28]. The authors reviewed the open-ended responses and developed line-by-line coding without a prescribed structure. The translation of responses into codes evolved into the first iteration of themes. The authors met, discussed the findings, and agreed upon the final descriptive thematic groups.

## 3. Results

Table 1 displays the demographics of the participating students including their age, gender, and school affiliation.

Four hundred and eight students participated in one of the two case simulations, and 386 students (94.6%) had matched pre- and post-test results from the surveys. The ICCAS scores as displayed in Table 2 showed significant improvement on pre- and post-test results, with 15.9% improvement overall, (*t* = 35.16; *p* < 0.001). Response time for the ICCAS pre- and post-surveys was under 6 min (mean = 5.35; 4.38); and response time for the pre- and post-open-ended surveys was under 7 min (mean = 5.22; 6.48). The questions were grouped and analyzed according to the competency domains of collaboration, roles and responsibilities, patient-centered approach, conflict management, and team functioning. Results indicate statistically significant improvements across each domain.

**Figure 1:**
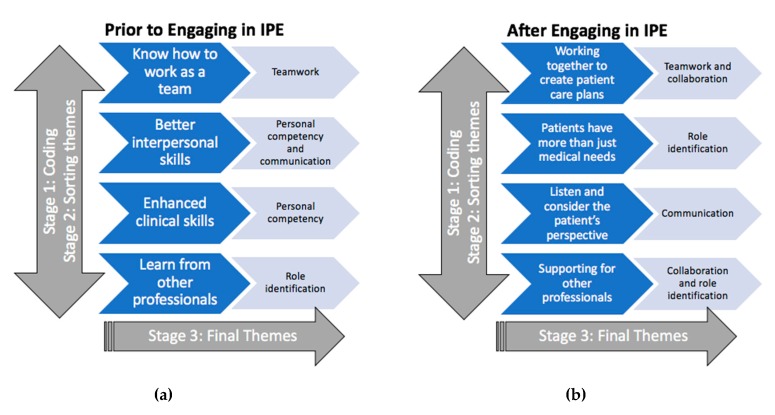
Results of thematic synthesis from open-ended questions: (**a**) prevailing themes from students’ responses prior to engaging in IPE; (**b**) prevailing themes from students’ responses after engaging in IPE.

A review of the open-ended surveys showed prevailing themes of collaboration, communication, teamwork, and role identification. The process is described in Figure 1 and shows the results of thematic analyses of student reflections before and after engaging in IPE. Approximately 167 students (43.2%) responded that they had prior IPE experience; these students were from the medical, nursing, or dental school. Students commented on the diversity of their teams and how this strengthened their care plan. A quote by one student exemplified the theme of collaboration: “I learned about the value of working as a team to come up with a cohesive plan of care for our patient. Relying on the expertise of different team members makes sure that your plan covers all the important aspects of the patient′s needs from social needs to medical needs.” Regarding communication, a student wrote the most important lesson learned was (to), “…try to take a couple of seconds to formulate my thoughts before answering the questions. Think from the patient′s point of view.” Another student wrote regarding communication, “We should improve in our communication skills such as choosing the right words when explaining the condition to the patient and family. Try to hit the point early and recognize the patient′s frustration and address it efficiently.” A greater number of students stated they found teamwork satisfying and responded they would rather wait for others’ input before acting alone after the simulation when compared with pre-simulation surveys. Regarding teamwork, one student wrote, “I think it was really good to have us all work together.” In a similar response, another student commented in the debrief they learned a great deal about insurance, support services, and the value of administrative services that may be provided for patients with little resources.

Most students stated that before the simulation, they had no prior knowledge of the role of a chief information officer or quality officer (roles played by the informatics student and public health student, respectively). Medical students were unaware of the function of a dentist in the inpatient setting. This provided an opportunity for dental students to share their knowledge and expertise. Students commented they gained a better idea of the training and roles of other students on the healthcare team and how to “utilize others’ skill sets to improve patient care”. For example, one nursing student commented how quickly the informatics student was able to navigate through the EHR, allowing them to find the error in the patient’s weight. Also, students reported the immediate feedback provided by the SPs was a valuable component to the exercise and helped them to better understand the concerns of the patient and of the patient’s family. Student teams were competent in their communication and teamwork skills as evidenced by faculty observation. We recognized the clinical students’ reflections remained focused on the patient and their communication with colleagues; further, the integration of students from public health and informatics did not detract from their self-assessed attainment of team competency.

## 4. Discussion

Our study found that by creating new clinical simulations that incorporated roles for public health and informatics students, students were able to observe and learn interprofessional teamwork from each other. Further, during the debrief and discussion post-simulation with IPE faculty, students were able to learn from faculty role models representing multiple disciplines within the patient care experience. Wilkes and Kennedy suggest, “students learn to practice as a team only when their instructors and mentors model the respect and trust for colleagues” [29]. Although the instruction was through simulation and not in a clinical setting, the implementation and post simulation debrief provided a level of faculty role-modeling which is not evident elsewhere in the curriculum. Though there were teams with medical and nursing students playing the roles of the chief information officer or quality officer when a non-clinical student was not available, we feel this was a valuable exercise. The clinical students needed to work with faculty advisors to learn other roles and put themselves in the position of another member of the healthcare team. The SPs were unaware of any substitutions and treated the students as the roles they represented, adding to the simulation experience.

The results of this study indicate that diverse teams of students, both clinical and administrative, who participate in interprofessional simulations can improve their awareness of the skills necessary for teamwork. Students were exposed to conflict and learned mutual respect through the inpatient medical error simulation, as the SP became irate with each of the team members. Resolving the conflict and backing up their peers allowed students to practice conflict resolution, humility, respect and mutual support—all of which are rarely taught in health science training programs [29]. In both patient cases, unexpected events (X-ray results and an irate patient) occurred halfway through the exercise and compelled the students to remain flexible to the changing environment in order to regulate their care plan. A commitment to the wrong direction at this stage would have signaled an error in the overall team functioning; conversely, teams who were able to adapt and display support for other members were commended. Students were also exposed to a change in clinical decision making, inclusive of health disparities; the team was required to foresee all possible outcomes of the patient’s prognosis including their financial status, ability to pay for their care, and social support. This step required the skills of both the clinical and administrative students. Reflections from the students indicate their appreciation of the various roles involved in the healthcare team. Dental students are specially trained to treat teeth as well as diseases of the mouth; further, they perform complementary preventive screenings necessary for overall health. Public health students have subject matter expertise in insurance, administration, and connections to other allied health sciences such as social work. Informatics students consider important hospital processes which include EHRs and other analytical workflows. Students were given a structured environment to share clinical rationale, ask for input, and seek joint problem solving; these are skills which contribute to a successful and collaborative team [30]. Further, both simulations allowed students to better understand the roles of their clinical and non-clinical peers, a recognized knowledge gap for many medical students [31]. Also supported in the results from the thematic analysis is a shift from personal competence (focusing on the individual) to collaboration (focusing on the team).

Future directions will include education of each team member’s role, such as an instructional video of a nurse practitioner, a chief information officer, and a chief quality officer, as these roles seemed to be to most misunderstood by students. We plan to collect and analyze differences within programs (e.g., BSN, MSN, and DNP; MS and PhD). The results may inform faculty as to the training needed for roles such as leader and team member that students eventually fill once in the working environment. We also plan to measure the retention of learned team skills by testing students in a clinical environment post-graduation.

## 5. Limitations

Although these cases have been implemented into the required curriculum at the medical, dental, and nursing schools as of July 2017, they are voluntary and not part of the required curriculum for the school of public health or informatics. As such, the majority of students in our study were from the schools of medicine and nursing, leaving many teams without an administrative or informatics student. Due to low participation from the disciplines of public health and informatics, it is not feasible to generalize these results. We did not collect data on the number of students enrolled in public health or informatics with prior clinical training but plan to add this question to future simulations. A greater distribution of students from all areas would be ideal in order to create a more realistic environment.

## 6. Conclusions

Our results suggest when a diverse body of clinical and non-clinical students participate in an IPE simulation, this may increase certain competencies for students across disciplines and may foster collaboration within interprofessional teams. Based on these findings, we recommend other university training programs adopt similar approach to including both clinical and non-clinical students. Future research and IPE simulations should also focus on including population health, telemedicine, and other relevant topics facing professionals and clinicians in healthcare.

## Figures and Tables

**Table 1:** Demographics of students participating in the interprofessional education (IPE) program (*n* = 408).

Demographic	Total, No (%)
Professional School Affiliation	
Medical School	198 (48.5)
School of Nursing	157 (38.5)
School of Biomedical Informatics	16 (3.9)
Dental School	22 (5.4)
School of Public Health	15 (3.7)
Gender	
Female	321 (78.7)
Male	87 (21.3)
Age	
20–29	216 (52.9)
30–39	133 (32.6)
40+	14 (14.5)

**Table 2:** Interprofessional collaborative competency attainment survey (ICCAS) results from completed pre- and post-test surveys (*n* = 386).

Competency Domain	Pre-Test Mean (SD)	Post-Test Mean (SD)	*p* Value
Communication	5.29 (1.59)	6.09 (1.23)	<0.001
Collaboration	5.21 (1.69)	6.11 (1.22)	<0.001
Roles and Responsibilities	5.29 (1.61)	6.11 (1.24)	<0.001
Patient-Centered Approach	5.10 (1.84)	6.06 (1.26)	<0.001
Conflict Management	5.57 (1.62)	6.20 (1.31)	<0.001
Team Functioning	5.00 (1.86)	6.01 (1.30)	<0.001
Total	5.26 (1.69)	6.10 (1.25)	<0.001
